# Notes and News

**Published:** 1886-12-18

**Authors:** 


### NOTES AND NEWS.

COLLECTED AND COMMUNICATED.

By command of the Queen a grant of seventy-five pounds worth of cast linen has been sent for the use of the little patients in the Victoria Hospital for Children at Chelsea, and similar presents to the London, St. Mary's, St. George's, and St. Thomas's Hospitals. Cast linen is always an acceptable present at hospitals, but it is surprising how few there are who bear in mind the value of such gifts.

At a meeting of the members of The Hospitals Association, held on Wednesday, the 15th inst., Sir William Mac Cormac, F.R.C.S., in the chair, Mr. Samuel Osborn, F.R.C.S., read an interesting paper on "Foundling Institutions at Home and Abroad," which was followed by a discussion to which we may refer again next week.

A meeting will be held at the Mansion House on the 20th inst., with the object of raising £100,000 on behalf of the Maintenance Fund of Guy's Hospital.

Mr. Frank H. Barendt, L.R.C.P.Lond., M.R.C.S.Eng., of University College, Liverpool, has just received the appointment of Senior House Surgeon to the Bootle Borough Hospital.

The endowed lectures at the Royal College of Physicians will be delivered in March; the Gulstonian lectures by Dr. Donald McAlister of Cambridge, on "The Nature of Fever"; the Croonian lectures by Dr. Broadbent, on "The Pulse"; and the Lumleian lectures by Dr. Priestly, on "The Pathology of Intra-Uterine Death."

The late Mr. Henry Boddington, the well-known Manchester brewer, has left some valuable legacies to the Lancashire charities. The Manchester Royal Infirmary and Dispensary receives £1,000; St. Mary's Hospital and Dispensary, Quay street, Manchester, £250; the Salford and Pendleton Royal Hospital and Dispensary, £250, and the Stockport Infirmary £150.

The bazaar at the Royal Concert Hall, Hastings, in aid of the East Sussex Hospital, to which we referred last week, was a grand success. The first day realized about £1,000, and on the two following days the results were equally satisfactory. The bazaar was so crowded that many persons failed to gain admittance. The inaugural ceremony was performed by Viscount Hampden, Lord Lieutenant of Sussex.

Attention has frequently been called in these columns to the necessity for adding to the incomes of hospitals by increased efforts on the part of hospital committees. University College Hospital shows excellent results in this direction, as the following returns will testify:—

| | | | | |
|---|---|---|---|---|
| 1884. Mansion House Meeting | - - - | £4,370 | 0 | 0 |
| „ Christmas Appeal | - - - | 1,008 | 0 | 0 |
| 1885. Festival Dinner | - - - | 3,482 | 0 | 0 |
| „ Christmas Appeal | - - - | 769 | 0 | 0 |
| 1886. Fancy Dress Ball | - - - | 1,502 | 0 | 0 |
| „ Bazaar (Two days) | - - - | 2,692 | 0 | 0 |
| Total | | £13,823 | 0 | 0 |

The amount contributed, in the Collecting Boxes in the Out-Patient-Rooms in 1885, amounted to £26 14*s.* 5*d.*; and, in addition, £29 were collected in boxes placed on the ward landings and elsewhere.

With reference to a paragraph which appeared in our last issue, respecting the new site for the St. Alban's Hospital, a correspondent writes:—"The rejection of Mr. Blundell Maple's offer proceeded rather from personal bias and an imperfect grasp of the necessities of the case, on the part of certain members of the Committee. The sole objections urged against Mr. Maple's site were the proximity of the Schools and the Church—although the school is fully sixty yards away and the objection of church-bells holds good even with the site which the Governors have chosen. By their ill-considered course, the institution loses a promised subscription of £200 from Lord Spencer, in addition to a gratuitous site from Mr. Blundell Maple. The one selected will cost £750, and the number of wealthy subscribers is certainly not "legion."

---

The collection at St. Michael's, Chester Square, invariably heads the list of church collections in aid of the Hospital Sunday Fund. This year the amount exceeded a thousand pounds, the average contribution "per head" being about thirteen shillings.

---

The following vacancies are announced this week, the amount of the salary being placed in each case after the name of the institution :—

**Honorary Medical Officers.**

Margate Infirmary. Two Surgeons, resident in Isle of Thanet.
Colchester General Hospital. Physician.

**Resident Medical Officers.**

Fulham Union. Assistant Superintendent.—£100, with board, etc.
James Murray's Royal Asylum, Perth. Assistant.—£70.
St. Luke's Hospital. Clinical Assistant.—Board.
Stone Lunatic Asylum, Dartford. Assistant. Doubly qualified.—£120, with board.

**Non-Resident Medical Officers.**

Western General Dispensary, Marylebone. Dispenser, fully qualified.—£80.
Chorlton Union, Manchester. District Officer and Public Vaccinator.—£110.

**Trained Nurses.**

Brighton Sanatorium. £22, with board, uniform, etc.
Stockport Infirmary. Two.—Uniform, etc.
Nursing Institution, Ryde. Several.—£22 to £30, with uniform.
Nursing Institution, Ryde. District.—£27, with uniform.

---

We have received an interesting letter from Miss Wilson, the secretary of the Workhouse Nursing Association, in which she says, "I find The Hospital is extremely popular amongst our nurses. I think a corner occasionally with suggestions for medical reading for nurses would be valuable, with the price and the name of the publisher of the books recommended." We shall be glad to adopt Miss Wilson's suggestion, and, with this object, to receive the names of authors and publishers, with the prices of any books of the description referred to, that our nursing readers may have found valuable for the purpose.

---

"Hospitalise" is the latest addition to our already vast *thesaurus verborum.* To "hospitalise," it seems, means to convert the unthinking to a knowledge of what our hospitals are doing in the noble cause of suffering humanity. To hospitalise the people expresses just what we want to do. The word, except for its hybrid composition, is not a bad one, and may possibly survive. However, we offer it to our readers with all due deference, merely acting on Captain Cuttle's advice, "When found make a note of."

The West London Hospital at Hammersmith is again in full work. The hospital has now 101 beds. Last year 1,269 in-patients were admitted, and 14,933 out-patients received attention. Some months ago, the managers greatly feared that they would have to close some of the wards of the hospital, and, despite the efforts which were then made to reduce the debt, there is still an incubus of over £7,000. The annual receipts average about £4,100, and the managers are now anxious to increase the income to £5,600.

---

Many of the children in our hospitals are just now waiting with pleasurable anticipations the toys which the subscribers to the *Truth* Toy Fund annually provide. To-day and Monday there will be an exhibition of all these pretty things at Willis's Rooms, which have generously been placed at Mr. Labouchere's disposal free of charge. The gifts to the fund this year are more numerous and more beautiful than ever ; some of the dolls would, we think, gladden the heart even of a little princess. "A Friend," who, in 1883 sent *Truth* 5,000 new sixpenny pieces, and 8,000 in 1884 and in 1885, has this Christmas forwarded 9,000 of these bright little silver pieces for distribution among the children. In this cold-hearted world of ours, it is pleasing to see so many warm hearts ; so many kind men and women who help to make happy at Christmas time those little children whose pleasure depends upon our bounty. "I love these little people ;" wrote Charles Dickens, of children, "and it is not a slight thing when they, who are so fresh from God, love us."

---

A lively little incident occurred at the meeting of the Metropolitan Provident Medical Association last week, touching the treatment of out-patients by hospital students. Mr. W. Bousfield, the Chairman of the Association, speaking of some of the evils arising out of the out-patient system, asserted that cases were very often treated by students who were not medically qualified. Sir Andrew Clark, who occupied the chair, expressed his entire dissent, and called on the House Governor of the London Hospital for an authoritative statement on the subject in connection with that institution. Mr. Nixon replied that such practice did not prevail at the London. For a little time the storm blew over, but, in the discussion later on, a young gentleman, whose name did not transpire, took occasion to reopen the burning question, supported Mr. Bousfield's contention, and called the chairman to task for his "dogmatic" assertion. The practice was, he said, common at his *alma mater*, and he added that he had himself, before he was qualified, attended, unassisted, hundreds of cases. Sir Andrew Clark observed that he spoke as to facts in regard to the London, whereupon the *quondam* medical student apologised to the chair, but observed that he was glad that the chairman limited his observation to the London. Mr. Bousfield, who had been looking up his "authority," then stepped up to the platform, and quoting from a pamphlet, said that only nine hospitals out of forty-eight which were asked the question, were able to say that out-patients were never attended by their students.

Miss Wood, the Matron of the Hospital for Sick Children, Great Ormond Street, read a highly practical paper last Friday, on "The Management of Infants and Children in Health and Sickness," at the Midwives' Institute, 15, Buckingham Street, Strand. The foundation of good habits in little children could, she said, be laid by watchfulness on the part of the nurse. Each infant brought into the world had peculiarities of its own, and these should be studied by the nurse. Miss Wood first dealt with babes in health. The nurse, or mother who carried the child safely through the perilous times of infancy, often did a greater deed than if she had carried it through a dangerous illness. The lecturess then pointed out the matters which primarily claimed attention, such as the proper warmth of the child, washing, air, food, etc. Speaking of children in sickness, Miss Wood said it was enough to make one's heart ache to see, at the hospital with which she was connected, how often the child was the victim of the vice and intemperance of its parents. In caring for the sick infant, the first rule should be to consult an experienced doctor, for skilled advice was necessary. Purgatives and aperients were not so much in favour as formerly. Their result might for a time be satisfactory, but the over-stimulation which they induced was disastrous in the long run. A warm bath, warm flannels, fomentations, etc., would generally bring about the desired effect. Miss Wood said at Great Ormond Street they waged war against the ordinary feeding-bottles with the elastic tube. She advocated the boat-shaped bottles, which had the advantage that the child could not take its food too quickly. After touching upon teething, infectious disease, and the evil which the consumption of alcohol by the mother wrought in the child, Miss Wood concluded by urging upon her hearers the importance of showing mothers how much easier it was to bring up children in a healthy natural way. If each could do that, even in the case of one or two little babes, she would have done a good work. On the proposition of Mrs. Perry, seconded by Mrs. Walsh, a cordial vote of thanks was passed to Miss Wood. A discussion followed.

---

The directors of Nairn Hospital held a meeting the other day to receive a deputation from the Sanitary Committee on the advisability of building a special fever ward, to supply the needs of both town and country, and thus to relieve the ratepayers from the burden of maintaining a separate fever hospital in each parish. The proposal was favourably received, and a medical committee forthwith appointed to procure plans and an estimate of the probable cost.

---

A special meeting of the subscribers to the Wellington Dispensary was held at the end of last month to consider the tendered resignation of Dr. Rider, who has discharged the combined offices of surgeon and secretary for the last forty years. When Dr. Rider first undertook these important duties, the institution was struggling under the burden of a large deficit in its finances. This deficit has not only been cleared off, but there is now a balance of nearly £400 in hand: a fact which testifies better than any words could do, to the ability and energy of the secretary. After some discussion, and many expressions of regret, Dr. Rider's resignation was accepted, and the application of Dr. Johnstone for the double appointment unanimously approved.

---

It is satisfactory to note that even in Wales, where the breach between Church and Dissent is widening year by year, the claims of the hospitals still have power to unite the opposing parties. The Vicar of Wrexham preaches a special sermon in aid of the Infirmary every year, and this year selected for the occasion the inauguration day of the new mayor, whose example of a visit to the parish church was followed by a very large number of his fellow-townsmen, without distinction of creed. After the sermon a collection on behalf of the Infirmary was made, when nearly fifty pounds was obtained.

---

The need of a cottage hospital for Abingdon, a town of 7,000 inhabitants, has been long felt, and this need is now supplied, thanks to the generosity of the Governors of Christ's Hospital, who gave the site, and that of Mr. T. C. Clarke, the late M.P. for the borough, who has paid the entire cost of erection. The hospital contains four wards, holding eight beds in all; and two bath-rooms, as well as convalescent, operating, and out-patient rooms. Two thousand pounds has been raised by subscriptions in the town and neighbourhood for the endowment, and an annual income of £150 has been guaranteed by certain local charities.

---

A four-days' bazaar in aid of the Children's Hospital at Bradford was opened in St. George's Hall in that town on the 1st inst. by the Mayor, Mr. Angus Holden; and we learn that £1,575 was obtained from the first day's sales, whilst donations to the amount of £1,655 had been previously received. The Children's Hospital has proved of great value, and the applications for admission of late have rendered extension of its accommodation necessary.

---

On the 2nd inst., the new Eden wing which has been added to the Durham County Hospital was formally opened by Lord Durham. There was a large attendance at the ceremony; and at the luncheon which followed, the High Sheriff of Durham (Mr. T. Richardson) compared the financial condition of the Durham with that of the Stockton Hospital. He said that the Durham Committee seemed to get plenty of donations, but very few subscriptions, whilst at Stockton the case was exactly reversed. The working men of the latter place took much interest in the hospital, and regularly paid in penny subscriptions, and the boys halfpenny ones, so that from £1,000 to £1,200 a year was realised from this source alone. There were eight representatives of the working men on the Stockton Committee, and he suggested that the same course should be pursued at Durham, as it was found a very great advantage in Stockton.

